# Hodgkin lymphoma involving the tonsil misdiagnosed as tonsillar carcinoma: A case report and review of the literature

**DOI:** 10.1097/MD.0000000000009761

**Published:** 2018-02-16

**Authors:** You Qin, Lijuan Lu, Yanwei Lu, Kunyu Yang

**Affiliations:** a Cancer Center, Union Hospital, Tongji Medical College, Huazhong University of Science and Technology, Wuhan; b Department of Molecular Medicine, Zhongshan School of Medicine, Sun Yat-Sen University, Guangzhou, Guangdong, China.

**Keywords:** Epstein–Barr virus, extranodal, Hodgkin lymphoma, tonsil

## Abstract

**Rationale:**

Primary Hodgkin lymphoma (HL) involving the tonsil is extremely rare. Only about 20 such cases with verification of biopsy and immunohistochemistry have been reported. Because of its rarity and unremarkable clinical presentation, a timely correct diagnosis is very challenging.

**Patient concerns:**

A 43-year-old man complained left tonsillar enlargement and painless masses in left neck, with night sweat. The clinical examination found a marked tonsillar asymmetry, with an enlarged left tonsil and ipsilateral cervical lymphadenopathy and a normal right tonsil.

**Diagnosis:**

The patient was initially regarded as tonsillar lymphoepithelial carcinoma.

**Interventions:**

The patient received a resection of left tonsil and left cervical masses and then was definitively diagnosed as HL (IIEB). He was managed by 6 cycles of chemotherapy (adriamycin, bleomycin, vinblastine, and dacarbazine) and radiotherapy to the Waldeyer ring.

**Outcomes:**

The patient has been disease free for more than 3 years after diagnosis.

**Lessons:**

As the reason of an extreme rare occurrence of HL involving the tonsil, doctors can easily misdiagnose the disease as tonsillar lymphoepithelial carcinoma. This case serves as a reminder important role of biopsy.

## 1 Introduction

Tonsillar tumors are relatively common primary carcinomas in head and neck, and unilateral tonsillar obvious enlargement with regional lymph node swollen often raises the suspicion of malignancy in tonsil,^[1]^ when infection have been excluded. Squamous cell carcinoma is the most common pathologic type of tonsil tumors followed by non-Hodgkin lymphoma (NHL). Rarer types, including Hodgkin lymphoma (HL), are easy to be misdiagnosed. We present here an unusual case of lymphocyte-rich classical HL (LRC) primarily involved the tonsil initially misdiagnosed as tonsillar cancer.

## 2 Case report

**Figure 1:**
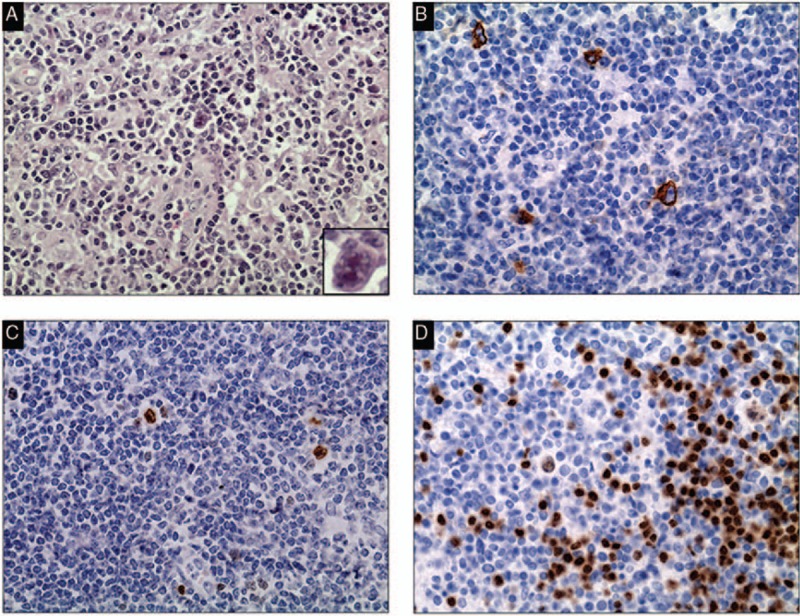
Pathological images of the left cervical lymph node. HE image shows scattered Reed–Sternberg cells within lymphocyte-predominant cellular infiltrates (A, ×40). IHC demonstrates that the neoplastic cells are positive for CD30 (B, ×40), Mum1 (C, ×40), and Pax5 (D, ×40). Inset typical Reed–Sternberg cell is shown in A (×400). HE = hematoxylin and eosin, IHC = immunohistochemical.

**Figure 2:**
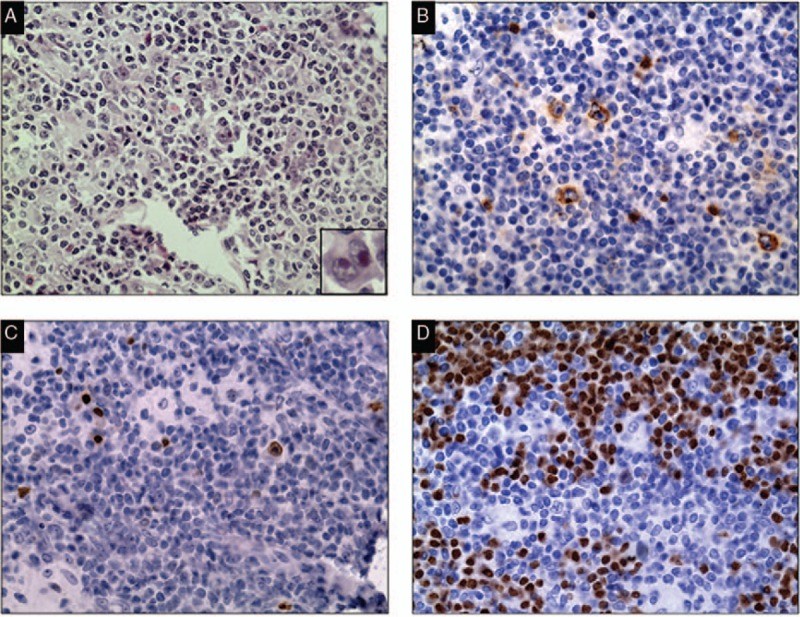
Pathological images of the HL involving tonsil. HE staining is shown in A (×40) and IHC of CD30 (B, ×40), Mum1 (C, ×40), and Pax5 (D, ×40) are also shown. Inset typical Reed–Sternberg cell is shown in A (×400). HE = hematoxylin and eosin, HL = Hodgkin lymphoma, IHC = immunohistochemical.

In November 2013, a 43-year-old man presented at local hospital with complains of left tonsillar enlargement and painless masses in left neck for about 1 year. He had no significant odynophagia. He had night sweat, but no fever or weight loss was noted. Medical history was rather normal, and he was a current smoker with 30 pack-year history. A clinical examination of this patient found a marked tonsillar asymmetry, with an enlarged left tonsil and ipsilateral cervical lymphadenopathy and a normal right tonsil. The rest of the physical examination was unremarkable. The ultrasound imaging of the neck demonstrated many cervical masses, and the biggest one of 39 × 25 mm is in the left side. He received treatment of antibiotics, but the enlarged tonsil and masses which had no response. Then fine needle aspiration cytology of this biggest lymph node was performed. Cytological smear showed a large number of lymphocytes under the vision, and some cells grew more active. He was diagnosed as tonsillar lymphoepithelial carcinoma, which is a kind of undifferentiated or poorly differentiated squamous-cell carcinoma with redundant lymphocytes infiltrated. Then a resection of left tonsil and left cervical masses was performed without surgical contraindication. Microscopic examination of the lymph node biopsy reveals partial effacement of the lymph node architecture. There is proliferation of large atypical mononuclear cells in a background of abundant inflammatory infiltrates. The mononuclear cells had hyperchromatic nuclei with vesicular smudged chromatin and prominent cherry red nucleoli, resembling Reed–Sternberg cells (Fig. 1A). These findings were more typical in the tonsil than the lymph node (Fig. 2A). A diagnosis of LRC was supported according to the biopsy and immunohistochemistry (IHC) analysis: CD20 (−), Fascin (+), CD21 (+), Ki-67 (+), Pax-5 (+), CD30 (+), CD15 (+), Mum1 (+), Bcl-6 (+), EMA (−), PD1 (−), TdT (−), Bcl-2 (−), CD10 (−), Cyclin D1 (−), Kappa (−), Lambda (−), and ALK (−). In situ hybridization for Epstein–Barr virus (EBV)-encoded small nuclear RNAs (EBER1) expression was negative. Due to the unusual location of HL, the original pathological specimens were sent to pathology experts of Peking University Third Hospital for a second consulting opinion to confirm the diagnosis. They got the same pathological diagnosis with such IHC analysis (Figs. 1 and 2): CD10 (−), Bcl-6 (−), CD20 (−), Pax-5 (+), Bcl-2 (−), Ki-67 (+), Mum-1 (+), Oct-2 (−), Bob-1 (−), and CD30 (+).

**Figure 3:**
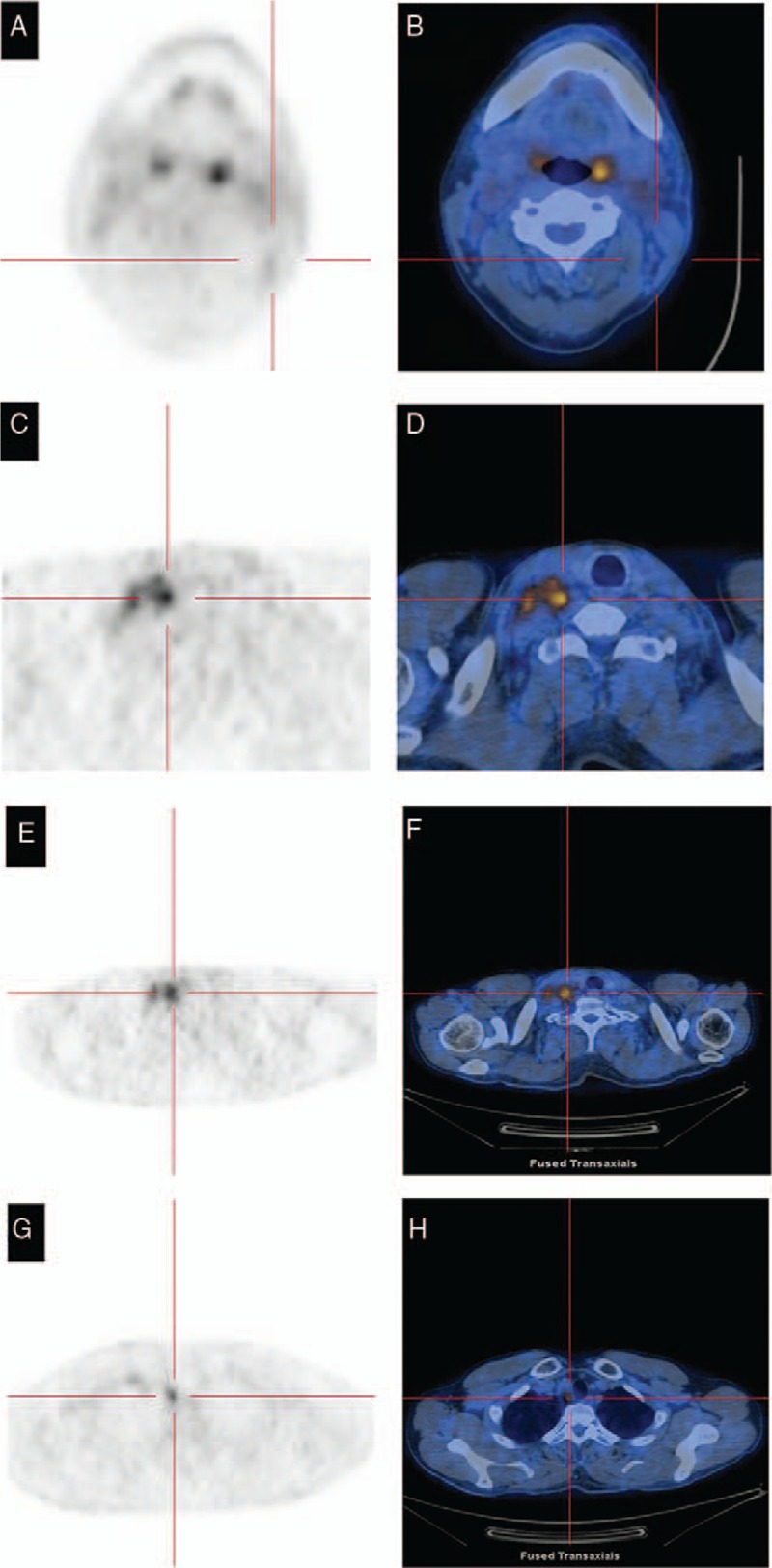
^[18]^Fluorodeoxyglucose positron emission tomography–computed tomography scan of the present lymphocyte-rich classical HL: fluorodeoxyglucose avidity were normal or slight increase in left tonsil and left cervical lymph nodes (A and B), because this scan was carried out after the resection of left tonsil and cervical lymph nodes. The standard uptake value (SUV) of fluorodeoxyglucose of multiple lymph nodes of the upper right clavicle (C and D), under right clavicle (E and F) and mediastinum (G and H) were high, with the maximum value 7.5. HL = Hodgkin lymphoma.

Additional ^[18]^fluorodeoxyglucose positron emission tomography–computed tomography (CT) scan revealed fluorodeoxyglucose avidity in multiple lymph nodes of the bilateral neck, upper and under right clavicle and mediastinum, with standard uptake value maximum was 7.5 (Fig. 3). No organ metastasis was detected. Bone marrow was negative. His disease was staged at IIEB according to the Ann Arbor classification system.

A chemotherapy regimen consisting of adriamycin, bleomycin, vinblastine, and dacarbazine (ABVD) was administered for 2 cycles, and computed tomography (CT) rescan showed a complete remission uncertain. After 2 more cycles of ABVD, the patient reached complete remission. Then 2 additional cycles of ABVD were followed. And the treatment was completed by involved field radiotherapy (IFRT) to the Waldeyer ring and involved lymph node fields using intensity-modulated radiation therapy. The total does was 30 Gy in 15 fractions (2 Gy per fraction, 5 fractions per week). Daily cone beam CT was done for image guidance and treatment verification. He was well-tolerated without suffering severe adverse reactions. This patient remains in good local control at the last follow-up visit at 18 months after diagnosis.

## 3 Discussion

The clinical treatment ways of different types of tonsil tumors are distinct. Although nonsquamous cell malignancies of tonsil are less common, they should not be ignored. The present case we reported was regarded as tonsillar cancer, and received unnecessary left tonsil and left cervical masses resection. The surgery increased not only physical and mental injury but also the medical burden to the patient, despite he was successfully managed by a sequential chemotherapy and radiotherapy. Doctors should be aware of these infrequent tonsil tumors to avoid the faulty treatment.

Tonsils are part of Waldeyer ring, which is a circular band of lymphoid tissue including nasopharynx, palatine tonsils, adenoids, and lingual tonsils and the base of the tongue. Lymphomas involve Waldeyer ring much less often, and the majority of those are NHL.^[2]^ HL of the Waldeyer ring is extremely unusual and accounts for only 1% of all malignant lymphomas of Waldeyer ring.^[3,4]^ According to Cionini et al,^[5]^ only 3.7% of the HL had involvement of Waldeyer ring. Here, we summarized the clinical characteristics of the published retrospective cases of HL involving Waldeyer ring in Table 1.

**Table 1:**
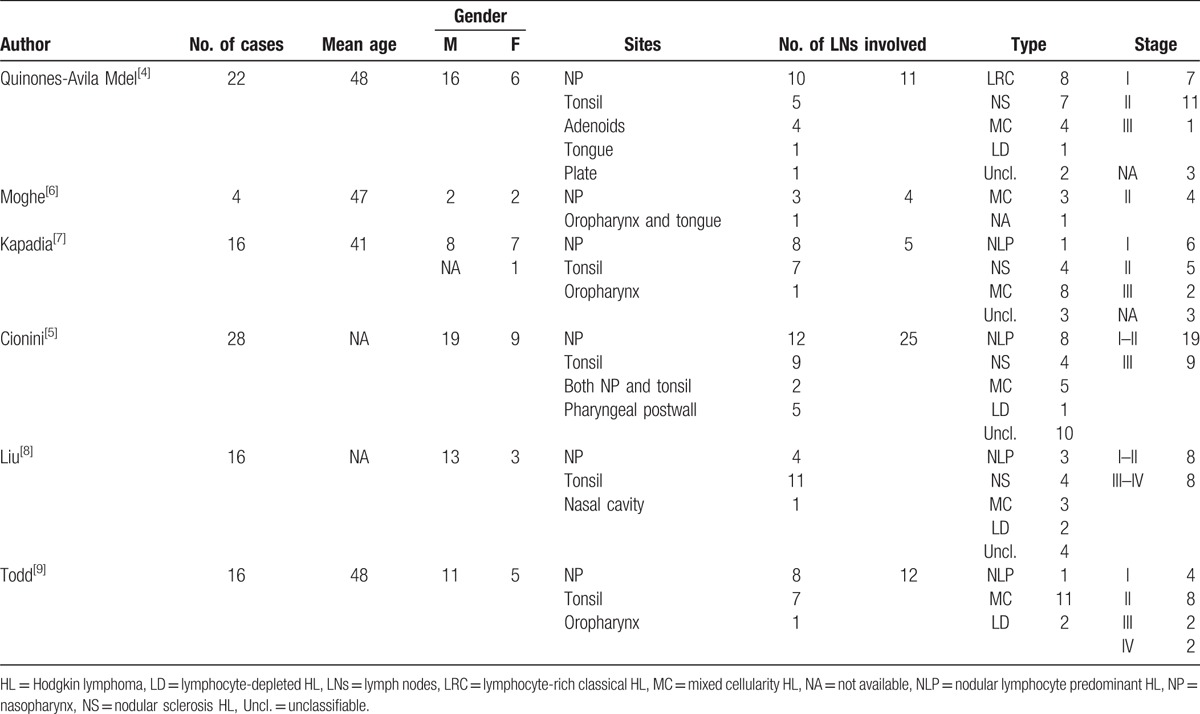
Series reports of HL involving Waldeyer ring in the literature: patient characteristics.

According to the table, the total number of patients was 102. There were 69 males (68.3%) and 32 females (31.7%), and the gender of 1 case was not available. The median age was 46 years. The disease was localized stage I–II to Waldeyer ring in 72 cases (75%), involved Waldeyer ring with or without cervical lymph nodes. And 24 cases (25%) were stage III–IV. This suggests that HL of Waldeyer ring used to appear mostly in men of early to middle age, and most of them are early stage. This is consisting with previous studies.^[4,7,10]^

We can learn from Table 1 that there are 47 HL cases located in nasopharynx, followed by 39 cases in tonsils. These account for 84.3% of all HL cases of Waldeyer ring. There have been some reviews of nasopharynx HL,^[11–13]^ but rare about HL involving tonsils. Approximately 60 cases of HL involving tonsils have been reported to the best of our knowledge; but many of them without verification of IHC.^[5,9,14]^ Here, the present case we reported and other 19 cases diagnosed by IHC were summarized in Table 2. There are also some isolated reports in Spanish.^[15,16]^

**Table 2:**
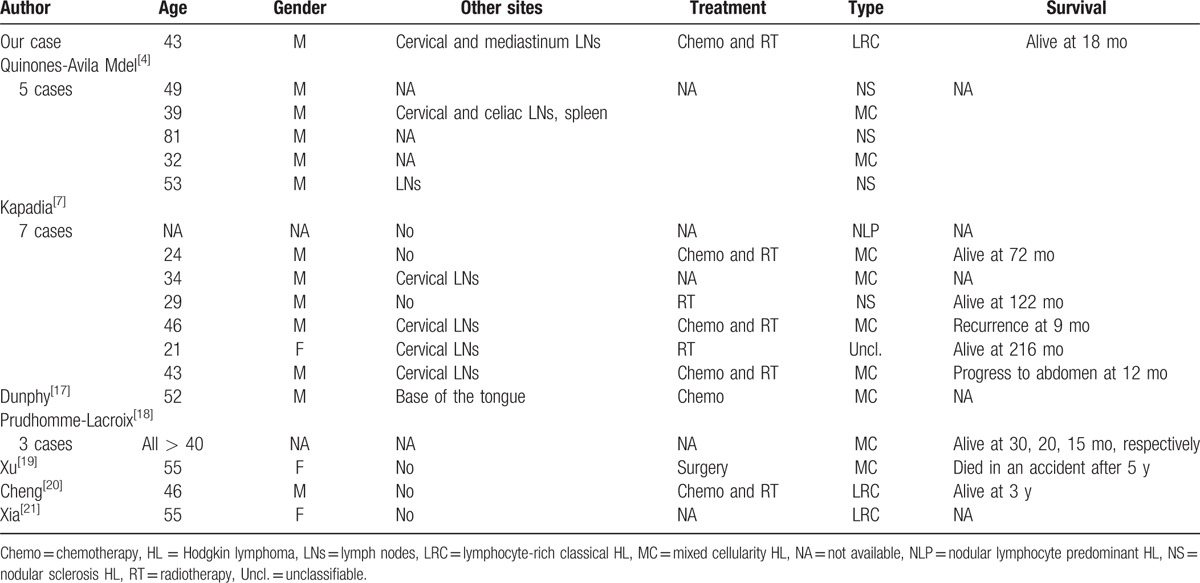
Cases reports of HL involving tonsil in the literature.

According to the available data of Table 2, the median age was 45.2 years, and 81.2% of them were male. Half of them with cervical lymph nodes involved. These characteristics are in line with ones of HL involving nasopharynx; 50% of these cases involved cervical lymph nodes. Histologically, 19 cases were clearly classified: nodular lymphocyte predominant, 1 (5%); lymphocyte-rich classical, 3 (16%); nodular sclerosis, 4 (21%); mixed cellularity, 11 (58%); and no lymphocyte depletion. This is consisting with the distribution in HL involving Waldeyer ring that mixed cellularity was the most common type.^[7,22]^ Present case was an LRC, a less common type.

However, Quinones-Avila Mdel et al^[4]^ suggested that lymphocyte-rich classical and nodular sclerosis types of HL account for higher proportion. According to their analysis, there may be 2 reasons for this discrepancy. One is that many cases of previous studies were diagnosed only on the basis of morphologic findings but were not confirmed by IHC, which is indispensable in diagnosis of initial HL primarily involving Waldeyer ring. The other one is that older lymphoma classification systems were used in previous studies.^[23]^ What's more, many cases classified in the past as mixed cellularity, nodular lymphocyte predominant, or unclassifiable HL might, in retrospect, be better considered the lymphocyte-rich classical type.^[24]^ Thus, we need more studies of HL involving Waldeyer ring diagnosed on IHC analysis and classified according to the new World Health Organization classification.^[25]^ The IHC results of 20 HL involving tonsils in the literature currently were reviewed in Table 3.

**Table 3:**
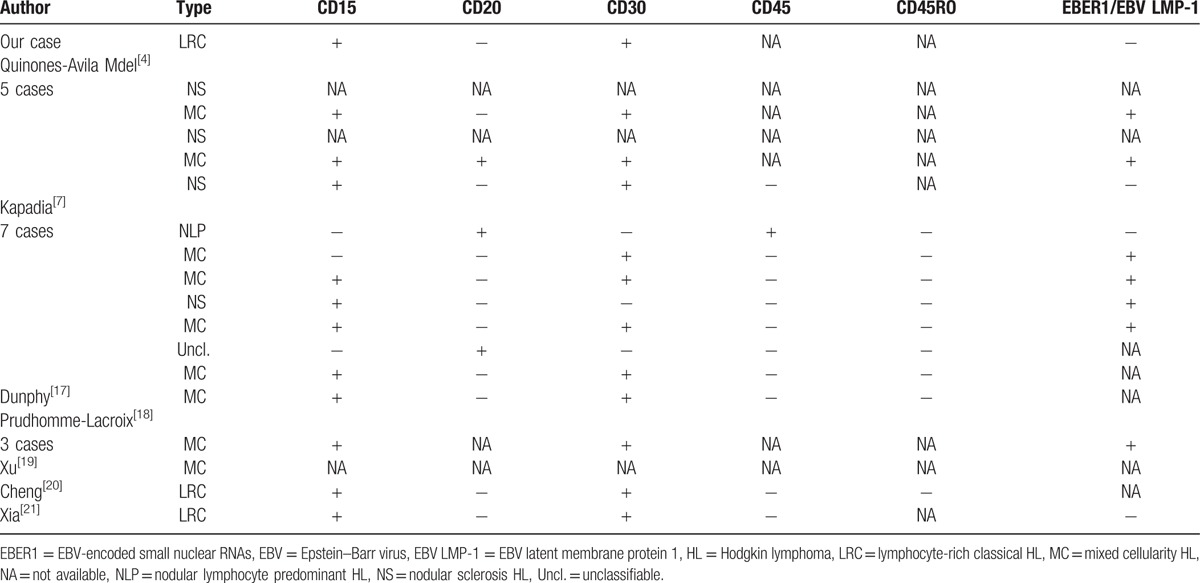
Immunohistochemistry findings in 20 cases of HL involving tonsil.

Both CD15 and CD30 were positive in 12 (80%) of 15 cases assessed, and they were both negative in the case of nodular lymphocyte predominant Hodgkin lymphoma (NLPHL) and the case of unclassifiable HL. But CD45 was positive in the NLPHL and negative for all other cases assessed. The neoplastic cells were negative for CD20 (n = 11, 79%) in 14 cases assessed.

EBER1 or EBV latent membrane protein 1 (EBV LMP-1) was positive in 9 (69%) of 13 cases assessed. Previous studies have reported that EBV can be detected in around 20% to 60% of classical HL.^[4,7]^ EBV may be involved in the pathogenesis of Hodgkin disease and not merely a silent passenger,^[26]^ especially those cases involving neck lymph nodes compared with HL cases involving none neck nodes.^[27]^ But the EBER1 expression of present case was negative. The evolution and progression of EBV negatives HL need to be further investigated.

The treatment of Waldeyer ring HL should be similar to that used in other HL localizations, which involve a sequential combination of chemotherapy and radiotherapy. ABVD is the appropriate standard regimen; and IFRT with target volume given as an intermediate dosage (25–40 Gy) targeting the Waldeyer ring and cervical lymph nodes should be the first line of treatment. In 2010, Iyengar et al reported the treatment and outcome of 34 HL of head and neck.^[10]^ Five received chemotherapy alone, 5 received radiation alone, and 24 received combination therapy. And 85% were disease-free at last follow-up. Despite the unique anatomic location of Waldeyer ring HL; it seems to not be of any special significance in the natural history of the disease.^[9]^ The standard HL protocols work effectively to promote disease-free survival.

## 4 Conclusion

HL involving the tonsil is extremely rare. Because of its rarity and unremarkable clinical presentation, a timely correct diagnosis is very challenging. Fine needle aspiration cytology is unreliable for making a diagnosis of this kind of disease. Therefore, biopsy of lymph node is necessary. The present study reveals that chemotherapy with subsequent radiotherapy is effective in the patient of HL involving the tonsil. To establish the best effective treatment strategy for this type of cancer, we need more reports like this to compare with different treatment methods and effectiveness.

## Acknowledgments

Special thanks are given to pathologists of the Union Hospital, Huazhong University of Science and Technology, and Peking University Third Hospital for their kindness of providing all the pathological images. The authors also thank Furong Lu from Department of Integrated Traditional Chinese and Western Medicine, Union Hospital, Huazhong University of Science and Technology, Wuhan for revising the language.
